# An Exploration and Confirmation of the Factors Influencing Adoption of IoT-Based Wearable Fitness Trackers

**DOI:** 10.3390/ijerph16183227

**Published:** 2019-09-04

**Authors:** Yu-Sheng Kao, Kazumitsu Nawata, Chi-Yo Huang

**Affiliations:** 1 Department of Technology Management for Innovation, The University of Tokyo, 7-3-1 Hongo, Bunkyo-ku, Tokyo 113-8656, Japan (Y.-S.K.) (K.N.); 2 Department of Industrial Education, National Taiwan Normal University, Taipei 106, Taiwan

**Keywords:** internet of things (IoT), wearable fitness trackers, technology adoption, modified delphi method, decision making trial and evaluation laboratory (DEMATEL), partial least squares (PLS)

## Abstract

In recent years, IoT (Internet of Things)-based smart devices have penetrated a wide range of markets, including connected health, smart home, and wearable devices. Among the IoT-based smart devices, wearable fitness trackers are the most widely diffused and adopted IoT based devices. Such devices can monitor or track the physical activity of the person wearing them. Although society has benefitted from the conveniences provided by IoT-based wearable fitness trackers, few studies have explored the factors influencing the adoption of such technology. Furthermore, one of the most prevalent issues nowadays is the large attrition rate of consumers no longer wearing their device. Consequently, this article aims to define an analytic framework that can be used to explore the factors that influence the adoption of IoT-based wearable fitness trackers. In this article, the constructs for evaluating these factors will be explored by reviewing extant studies and theories. Then, these constructs are further evaluated based on experts’ consensus using the modified Delphi method. Based on the opinions of experts, the analytic framework for deriving an influence relationship map (IRM) is derived using the decision-making trial and evaluation laboratory (DEMATEL). Finally, based on the IRM, the behaviors adopted by mass customers toward IoT-based wearable fitness trackers are confirmed using the partial least squares (PLS) structural equation model (SEM) approach. The proposed analytic framework that integrates the DEMATEL and PLS-SEM was verified as being a feasible research area by empirical validation that was based on opinions provided by both Taiwanese experts and mass customers. The proposed analytic method can be used in future studies of technology marketing and consumer behaviors.

## 1. Introduction

The internet of things (IoT), also called the internet of everything or the industrial internet, is a new technology paradigm envisioned as a global network of machines and devices capable of interacting with each other [1]. Due to advances in internet technology, the connected objects (e.g., smartphone) and information networks enable communication between people, machines, or objects. The IoT can be conceptually defined as a dynamic global network infrastructure with self-configuration capabilities [2]. In the IoT paradigm, many of the objects that surround us will be on the network in one form or another [3]. The IoT has been regarded as an important component of information communication technology (ICT) and media industry. Moreover, IoT has been hailed as being part of the foundation for industry 4.0 due to the possibility that it can generate drastic changes to existing industries and business models [4]. Thus, the IoT is one of the most important areas of future technology and is gaining vast attention from a wide variety of industries [1]. The IoT will be the next dominant form of information technology (IT), which will significantly influence the welfare of human beings from the aspects of healthcare, supply chain management, energy saving, smart control, intelligent building, and product life cycle management. 

A smart device is an electronic device that can be used to address various tasks in our daily life via different wireless protocols such as Wi-Fi, Bluetooth, and RFID [5]. Smart devices can fulfill daily needs and thus have become very popular during recent years. Due to the progress in IoT technology as well as computation techniques, IoT-based smart devices, which integrate physical object connection, cloud computation, machine learning techniques, and other data analysis approaches, facilitate convenient use of smart applications more than ever before. For example, doctors can inspect a patient’s heart rhythm by remote monitoring using IoT-based smart devices. Farmers can also use IoT-based smart devices to optimize the irrigation efficiency of crops. 

According to market analysis, 20.8 billion units of IoT devices will be installed worldwide and total spending on IoT endpoints and services will reach almost $3 trillion in 2020 [6]. In recent years, IoT-based smart devices have penetrated a wide range of markets, including connected health, smart home, and wearable devices. Among the IoT-based smart devices, wearable fitness trackers are the most widely diffused and adopted IoT based devices. Such devices can monitor or track the physical activity of the person wearing them. According the latest report published by P&S market research, the market of global wearable fitness trackers is expected to reach the revenue of $48.2 billion by 2023. The growth is led by increasing use of fitness tracking apps, rising demand for wireless and continuous health monitoring devices, thriving awareness about obesity, and increase in disposable income.

Facing such a trend, exploring business opportunities for the applications of IoT-based wearable fitness trackers will be necessary for the development of new consumer electronic products. Understanding the drivers and features that influence people’s decisions to adopt IoT-based wearable fitness trackers will be indispensable for designing an appealing IoT-based smart device. Nevertheless, accurately predicting technology usage behaviors is not easy [7]. Such predictions always involve complex and uncertain factors that are difficult to identify. Thus, an accurate prediction model for exploring technology usage behavior will be necessary. 

However, previous studies of technology acceptance behavior mainly focused on general mobile devices [7,8,9,10,11,12,13,14,15,16]. Although society has benefitted from the conveniences provided by IoT-based wearable fitness trackers, few studies have explored the factors influencing the adoption, users’ being aware of, embracing, and fully utilizing, of such technology. Furthermore, one of the most of concerning issues nowadays is the large attrition rate of consumers no longer wearing their device. Current business models are built on technology push and therefore do not succeed in matching the technology to consumer needs. Previous studies have either focused on the technological features or adoption potential of wearables. Yet, little is known about the elements leading to the adoption in general, and the attrition of wearable fitness trackers especially. According to Renaud and Van Biljon [17], technology adoption is a process—starting with the user becoming aware of the technology, and ending with the user embracing the technology and making full use of it. Therefore, the purpose of the paper is to identify the key determinants from a consumer perspective that lead to dissatisfaction and, eventually, wearable attrition.

Many academic scholars and social psychologists have proposed theories regarding technology adoption behavior, including the theory of reasoned action (TRA), the theory of planned behavior (TPB), the technology acceptance model (TAM), the diffusion of innovation (DOI), technology readiness index (TRI), and the unified theory of acceptance and use of technology (UTAUT). So far, these theories have been widely accepted and applied in various fields such as behavior science, system engineering, management, computer science, and education. Drawing on insights from the extant technology adoption literature, one objective of this article is to define an analytic framework for exploring the factors that influence the intentions of users that adopt IoT-based wearable fitness trackers. To establish a theoretic model, appropriate variables were defined based on past literature towards technology adoption. Conventional empirical studies of technology adoption typically inferred consumers’ intentions of technology usage based on observed variables. These inferences were statistically confirmed by using methods such as regression analysis or structural equation modeling (SEM). The path relations hypothesized in analytic models are often based on theoretical models or results derived from an exploratory factor analysis. The effectiveness of these methods has been verified and can be generalized to other research fields and objectives. However, large sample sizes are usually needed to fulfill the requirements of traditional statistical approaches. Meanwhile, modeling procedures are always time-consuming. Furthermore, the paths constructed based on results of literature reviews may not be sufficiently comprehensive to reflect real-world problems. Therefore, a novel analytic framework would be helpful for solving the problems practitioners face.

To resolve the above-mentioned problems, this work proposes a novel analytic framework consisting of the decision-making trial and evaluation laboratory (DEMATEL) and the partial least squares (PLS) SEM approach. Instead of path modelling based on literature review results, the proposed framework introduces expert opinions based on the DEMATEL method to construct the influence relationship map (IRM). The derivation of the IRM can be further confirmed by using the PLS-SEM. Compared to the traditional covariance-based SEM (CB-SEM), the PLS-SEM has better predicting effects in both reflective models and formative models [18]. The PLS-SEM is also an appropriate method for this research, which makes minimal demands on complex models (with many constructs and many indicators) and residual distributions [19]. Thus, the PLS-SEM is very suitable for this work. The proposed analytic framework can address the above-mentioned problems, which are frequently found in traditional path construction and analysis procedures. Using the novel analytic procedure, the intentions of users who adopt a specific technology in general, and the IoT-based wearable fitness trackers in particular, can be inferred based on the opinions provided by a group of experts and then confirmed by mass customers.

The purposes of this work are two-fold. The first purpose is to establish an evaluation framework for exploring the factors that influence the adoption of a general novel technology and IoT-based wearable fitness trackers by users, from the perspective of experts. Then, the paths derived from experts’ opinions using the DEMATEL are confirmed by the opinions of mass users using the PLS-SEM. In general, the analytic framework involves several steps, as follows. First, aspects and criteria suitable for our analytic framework are derived from literature review results. Second, the aspects are evaluated and confirmed by experts using the modified Delphi method. Third, the IRM is compared to each derived aspect and criteria and modeled using the DEMATEL method based on experts’ opinions. Fourth, the IRM is further confirmed using the PLS-SEM method. Finally, using the proposed framework, the empirical analysis is verified based on survey results of Taiwanese mass customers that use smart wearable devices.

The remainder of the paper is structured as follows. In Section 2, the literature on technology adoption is reviewed. The evaluation aspects for predicting users’ intention for technology adoption are collected. Additionally, the development and related knowledge of IoT-based wearable fitness trackers are also reviewed. In Section 3, the proposed analytic framework based on the DEMATEL and the PLS-SEM is demonstrated. In Section 4, an empirical study case is used to demonstrate the feasibility of the proposed analytic framework. Section 5 discusses the research findings, managerial implications, and advances in research methods. Section 6 contains conclusions, research limitations, and future research.

## 2. Literature Review

In the IoT era, the connection of wearable fitness trackers to the Internet will influence the daily life of most people due to enhanced convenience and efficiency of performing activities and tasks. Therefore, understanding the factors that influence users’ intentions and behaviors of adopting and using IoT-based wearable fitness trackers is an interesting and important issue. Studies of technology adoption have been discussed in a wide range of domains over the past few decades since the proposal of TAM by Davis, et al. [20]. To explore the factors influencing the adoption of IoT-based wearable fitness trackers, related theories are reviewed in this section. Then, possible factors influencing the intention and behavior toward the use of IoT-based wearable fitness trackers are derived.

### 2.1. Wearable Fitness Trackers

In our daily lives, people can easily access the internet for browsing the web, exchanging data and information with other people in terms of real time data refresh, using multimedia content and services, working on projects, reading the latest news, using social networking applications, and many other tasks [21]. With increased developments in cloud computing services and the internet, more and more applications will be launched to fulfill people’s lives. Most developments in technologies and applications that allow machines and smart objects, including radio frequency identification (RFID) tags, sensors, actuators, PDAs, and smartphones, as well as virtual objects in cyberspace, such as data and virtual desktops on the cloud [22], to communicate and coordinate are established via the internet. In this situation, interactions between machines or machine and human are considered to be a type of IoT. 

Various kinds of smart devices based on IoT functions have been developed and commercialized. These devices collect, analyze, and distribute data such as air quality, rescue operations, and face recognition. Users of IoT-based smart devices can be classified into three main groups: consumers, enterprises, and industrial. From the perspective of consumers, IoT-based wearable fitness trackers are defined as wearable devices with IoT functions that can be attached to the human body as an accessary or embedded into clothes as external devices [23]. Examples of consumer IoT-based wearable fitness trackers are smart wristbands, smart trackers, and smart helmets. From the enterprise perspective, IoT-based smart devices can be tools used for meetings. For example, smart sensors placed in a conference room are able to automatically adjust the temperature and lighting, depending on the situation. From the industrial perspective, IoT-based wearable fitness trackers can function as a security system and can be used in traffic monitoring, production control, and diagnoses. 

Wearable fitness trackers (also known as activity trackers) are IoT-based devices that can monitor or track the physical activity of the person wearing them. These devices are typically worn like wristbands. Activity trackers can monitor activity parameters such as the number of steps taken in a specific time period, distances covered, average speed, and calories burned. Some fitness and activity trackers can also monitor heart rates and sleep quality. The tracking results can provide a good picture of the wearers’ health conditions. Some wearable fitness monitors can further support the definitions of daily fitness goals and demonstrate the progress made in fulfilling those goals. Such features enable people to be accountable for their daily fitness goals and help those users improve their health status. Smartwatches are an advanced version of fitness trackers, providing many more features than fitness trackers. 

In this research, the Xiaomi wristband will be the focal device used to analyze users’ adoption intentions and behaviors. The Xiaomi wristband has several features, like monitoring users’ daily fitness levels and tracking their sleeping performance, as well as notifying them when they should go to bed and wake up. The sleep performance feature can help users understand their health status and improve the quality of their sleep based on the data collected from the wristband. The Xiaomi wristband can also track users’ fitness activities over time, alert them to incoming calls, and notify them of important meetings. All these features are mainly based on the Xiaomi wristband based on IoT. In the past few years, the Xiaomi wristbands have been improved, and are currently one of the more popular IoT-based fitness trackers in the world. Due to the wide adoption of Xiaomi wristbands in the Taiwanese market, this research adopts the Xiaomi wristband as a target device to analyze users’ adoption behaviors toward the IoT-based wearable fitness trackers.

Related IoT-based wearable fitness trackers significantly affect the development of various smart applications, but also influence our daily lives. Such devices will become dominant tools in the near future that will facilitate development of smart applications, especially those for individual use. Therefore, in this paper, we focus on the viewpoint of an individual to explore and predict the usage behaviors of IoT-based wearable fitness trackers.

### 2.2. Technology Adoption

In the past several decades, with technologies evolving and merging all the time in this dynamic world, more and more theoretical frameworks for exploring users’ behavior in the adoption of novel technology have been presented. These theories include the DOI [24], the TAM, the TRA [25], the TPB [26], the TRI [27], the UTAUT [28], and the extension UTAUT2 [29]. Scholars defined these models to explore the reasons why users adopt or accept specific products and technologies. Thus, the factors influencing people’s acceptance of novel technologies or products can be extracted from these existing models. 

The traditional TAM model has two determinants: perceived usefulness (PU) and perceived ease of use (PEU). PU is defined as the extent to which a person believes that using some specific technology will enhance his/her job performance [30]. The PU aspect is mainly leveraged for evaluating an individual’s perception of whether the desired goal can be achieved by using some specific technology. Nielsen [31] defined usefulness as representing whether some specific technology can be used to achieve desired goals. Usefulness is a crucial concept for evaluating the practical adoptability of some specific technology, such as IoT-based wearable fitness trackers. These two notions are similar by emphasizing the actual utility of technology adoption. Thus, PU and usefulness are used interchangeably, since they both represent people who perceive whether a goal can be satisfied by adopting a specific technology [32]. Conversely, PEU describes the degree to which a person believes that using a technology will be effortless [30]. Nielsen [31] argued that users’ evaluation of the necessary effort in using technology is associated with their ability to use functional components of related technology. The definition of this concept is how well users can use functionality and which can be named as “usability” or “technology usability”. The PEU and usability indicate that something works well and that a person with average capability can use the technology for the intended purposes without frustration. Both PU and PEU determine users’ attitude toward adopting and using some specific technology or product. Most extended models for predicting human behavior as it relates to technology adoption often keep two important beliefs in the model. The UTAUT model is an example for using two important determinants. The concepts of performance expectancy and effort expectancy in the UTAUT model are very similar to the PU and PEU determinants in TAM. In other words, these two beliefs have a significant impact on the technology adoption model.

As the TAM-related models have demonstrated robustness and effectiveness [33,34,35,36,37,38,39], researchers often employ such models as theoretical foundations for analyzing adoptive behaviors of novel technology, including mobile banking services [10], smartphone adopting behavior [7], mobile learning [40], smart watch [33], and cloud computing technology [41]. For example, Palau-Saumell, et al. [42] utilize the SEM method to examine the adoption of mobile applications for restaurant searches and reservations by different level of users’ age based on the UTAUT2 model. Duarte and Pinho [43] used PLS-SEM and fuzzy-set qualitative comparative analysis to examine mobile health adoption. Tavares and Goulão [44] explored usage behavior of electronic health record portals by using the UTAUT model. Shaw and Sergueeva [45] used a modified UTAUT2 model where perceived value replaced price value to examine the mobile commerce adoption in Canada by SEM method. Recently, Raut and Priyadarshinee [46] proposed a three-stage research process to investigate factors influencing users’ adoptioon of cloud based computation. First, the SEM method was used to model a path relation network. Next, the ANN approach was utilized to derive the weights in each path relationship. Finally, the ISM method was used to identify important criteria. 

Though TAM-based models and derivations have been widely adopted for analyzing consumers’ behaviors toward adopting novel technologies, only a limited number of studies on users’ adoptions of IoT-based wearable fitness trackers have been performed. IoT-based wearable fitness trackers play important roles on a daily basis in consumer electronics. Therefore, this research aims to derive factors that can influence users’ adoption of IoT-based wearable fitness trackers and further define a causal relationship model among identified factors.

### 2.3. Model Development for Deriving Factors Influencing the Adoption of IoT-Based Wearable Fitness Trackers

Over the past several decades, TAM-based theoretical frameworks have been widely used for analyzing consumer behaviors toward novel technology and other adoptive behaviors. However, previous studies found the TAM-based models are incapable of providing consistent and superior explanations for behavioral predictions [47]. Due to this reason, a growing body of work has focused on expanding the original TAM with other models and variables to investigate factors that influence use of novel technology. Most studies have demonstrated the strength of such integrated models, which can derive better explanations for users’ adoptive behavior toward novel technology [15]. IoT-based wearable fitness trackers are novel technology being characterized with network externality, personal characteristics, and relevant technology knowledge. Therefore, an extended model based on the TAM and other predictive factors will be more suitable for understanding users’ intention to adopt IoT-based wearable fitness trackers. Accordingly, feasible aspects are proposed and will confirm the appropriateness of this research. These aspects include performance expectancy, user innovativeness, network externality, domain specific knowledge, perceived technology utility, perceived usability, adoption intention, and usage behavior. Through these factors, a causal framework is modeled based on the perspective of experts. Then, the effectiveness of the causal relation map for predicting the intention of users to adopt IoT-based wearable fitness trackers is further confirmed based on the opinions of mass customers. Following this, the constructs are introduced.

#### 2.3.1. Perceived Usability

Usability is associated with two constructs: ease of use and ease of learning [31]. Compared to TAM-related models, perceived usability is equivalent to perceived ease of use and effort expectancy. Both concepts represent the degree to which people believe that using a technology would be effortless [48]. In the context of technology adoption, perceived usefulness and perceived ease of use are the most important determinants for analyzing technology usage behavior and behavioral intentions [48,49]. Furthermore, usability can be leveraged as a critical factor for exploring the relationship between usage behavior and technology adoptive intentions. To accurately explain the concept of usability, Nielsen [31] proposed five attributes, including efficiency, learnability, memorability, errors, and satisfaction. Furthermore, perceived ease of use (or perceived usability) has been verified to be a positive impact factor on perceived usefulness [32]. The easier it is to use a novel technology, the more useful it will be. Based on these conceptual definitions, perceived usability can influence the adoptive intentions and usage behavior of users for a novel technology. In this research, we use usability to mean perceived usability, based on the study by Lacka and Chong [32]. Perceived usability in this study represents the perception of whether IoT-based wearable fitness trackers are able to satisfy the usage needs of users. Also, perceived usability will serve as an essential aspect for exploring the adoptive intentions of IoT-based wearable fitness trackers users.

#### 2.3.2. Performance Expectancy

Performance expectancy is an important part of technology adoption. Performance expectancy is defined as the extent to which a novel technology can provide benefits to users in performing daily activities [29]. With IoT-based wearable fitness trackers, usage needs can be easily fulfilled over the internet via wearable fitness trackers. The largest difference between IoT-based wearable fitness trackers and common mobile devices is the perception of users regarding whether IoT-based applications, such as health care monitoring, or smart control, can be used. IoT-based wearable fitness trackers will be capable of facilitating users’ activities more conveniently and will accomplish users’ needs more easily. Thus, IoT-based wearable fitness trackers will play a big role in the future. In addition, TAM-related models demonstrated that performance expectations positively influences adoption and usage intentions of users [50,51]. Based on this literature review, performance expectations are a pivotal factor for understanding users’ adoption intentions and usage behaviors.

#### 2.3.3. Perceived Utility

Perceived utility is an indispensable feature for exploring users’ intention to adopt and use a novel technology. Perceived utility (proposed by Nielsen [31]) evaluates whether a specific system or technology can fulfill the needs of customers. Perceived utility assesses whether the technology and its functional element can fit certain tasks [32]. In general, the success of a specific technology is mainly due to its unique design or functionalities. For example, users may consider IoT-based wearable fitness trackers to be especially useful since they provide useful functions and applications. In other words, the functionalities may increase users’ intentions to use a specific technology [52,53]. That is, increased perceived utility can have a positive influence on performance expectancy and users’ intention to adopt a specific technology. In this research, perceived utility represents the users’ perception of whether IoT-based wearable fitness trackers can be used to achieve daily activities.

#### 2.3.4. Network Externality

Network externality is defined as changes in benefits that a consumer derives from goods when the number of people consuming the same kind of goods changes [54]. In consumer product market, network externalities play an important role, as the effect derived from network externalities can bring about more benefits and profits for companies [55]. For IoT-based wearable fitness trackers, more benefit is achieved as more users adopt the device. As more people use such devices, more utility will be generated [56]. In general, network externality can be divided into two categories: direct and indirect. Direct network externalities are based on the number of participants in a given network [57]. Many studies demonstrated the effect of network externality on technology adoption [58]. That is, more people will try to adopt a specific technology, since they perceive that the number of people using the novel technology is increasing. Indirect network externalities demonstrate an increased sense of user value from using a product or technology, because such effects will be increased as the number of relevant complementary products increase. For these reasons, we aim to derive the relationship between the number of users of IoT-based wearable fitness trackers and other variables in this research.

#### 2.3.5. User Innovativeness

Rogers Everett and Schoemaker [59] defined innovativeness as the extent to which a user adopts a particular new product earlier than other people. Agarwal and Prasad [60] defined innovativeness as the willingness to try new information technology. Innovativeness can be understood as being a function of the dimensions of human personality [61]. Bruner, et al. [62] further defined user innovativeness as the propensity of specific groups of people to take risks. These people take chances and embrace new things and are quite willing to address situations that have high levels of uncertainty.

In the context of technology and innovation adoption, user innovativeness is considered to be an essential driver in adopting innovation [63]. The concept of user innovativeness is similar to the lead user. A lead user is defined as a user who has innovativeness traits and desires to accept new technology and products. The lead user based method was proven to be capable of providing the highest potential for creating commercially attractive and highly novel innovation [7,64]. In other words, the lead user method usefully explains users’ intentions and usage behavior. In addition, past studies showed that user innovativeness has a significant relationship with behavioral intention [65,66]. User innovativeness plays a key role in new technology adoption and innovation success. Consequently, users’ receptivity toward taking chances or trying new things should indicate people’s desire for innovativeness with regard to a specific technology [67], such as IoT-based wearable fitness trackers.

#### 2.3.6. Domain Specific Knowledge

The concept of domain specific knowledge was adapted from the concept of technology awareness [68]. Based on the definition, technology awareness encompasses users’ knowledge and understanding of a specific technology or product [69]. Domain specific knowledge indicates that users have sufficient knowledge and understanding for adopting a particular technology. Many studies have been performed that explore the relationship between technology awareness and behavioral intentions of adopting a specific technology [70]. Previous empirical studies imply that when users have sufficient domain knowledge of a particular technology, they will be willing to adopt this technology [71]. The relationship between domain specific knowledge and usage behavior was demonstrated by Bardram and Hansen [72]. User adoption of network-connected devices, such as in-home smart appliances and wearable technology, is increasing. It is predicted that more and more IoT-related products and services will soon emerge and change our lives. For potential consumers and current users, adopting such new technology will generate unexpected changes that will make people feel anxious. Thus, understanding the feasible applications of IoT and how IoT appliances can be used correctly and effectively will assist people to deal with potential uncertainties created from the use of IoT-based wearable fitness trackers. For this reason, awareness of domain specific knowledge by users in the market is important for IoT-based wearable fitness trackers adoption. For example, Persaud and Azhar [73] stated that consumers will possibly reject a particular mobile service through mobile appliances if they have little trust in it, and may not be aware of a technology product or service. A study by Chang, et al. [74] argued that smartphone users are willing to believe a social networking service (e.g., Facebook) and keep using it even with the perceived risks of Facebook usage. This is due to the users having sufficient knowledge and belief in the reliability of using Facebook. As IoT-based wearable fitness trackers represent a fairly new technology, fully understanding the development and application of IoT services may facilitate adoption of new technology. For these reasons, domain specific knowledge is an important variable that can be used to explore the adoptive intentions and usage behaviors toward a particular technology.

#### 2.3.7. Adopting Intention

Theorists and behavior analysts have broadly explored user behaviors and formed a consensus opinion on adoptive intentions that can be regarded as a dominant factor in the use of IT and technology products [7,29,33,41,48,75]. Adopting intention refers to the degree to which a person has formed conscious plans to perform or not perform some specified future behavior(s) [76]. Several studies proved that adoptive intention positively correlates with perceived usefulness and perceived ease of use. In other words, adoptive intention can serve as the basis for developing a behavioral research framework for novel technology. 

For example, the TAM and TPB models employed behavior intention as one aspect for predicting consumers’ acceptance of technology. Hybrid frameworks of technology acceptance research also utilize behavioral intention as an aspect of analytic framework (e.g., [51]). Theoretically, most analytic models of technology adoption study why people accept a new technology, product, or service, and study the relationships between users’ adoptive intention and other aspects that are integrated into the analytic framework. Practically, the IoT-based wearable fitness trackers are a novel technology. R&D and marketing managers of IT firms must understand exactly what reasons and features influence consumers’ adoption of such technology. Given these views from the literature, this research investigates the factors that affect users’ behavioral intention toward IoT-based wearable fitness trackers. The feature of adoptive intention is used for modeling the analytic framework.

#### 2.3.8. Usage Behavior

The concept of usage behavior has been validated and used in many studies for explaining new technology adoptive behavior by users [77]. To understand why people adopt or use a particular technology, “usage behavior” is always seen as an essential construct for developing predictive models. For example, Davis [30] applied the TAM model to computer technology usage behavior. In Davis’ study, usage behavior as a dependent variable is used to explore relationships among dominant variables such as perceived ease of use, perceived usefulness, and intention to use. Although usage behavior has been extensively used in the context of technology adoption, these past studies focused on the viewpoints of mass users. That is, very few studies have tried to understand the perspectives of lead users in adopting a particular technology. In technology adoption fields, using both perspectives from lead users and mass users may be a better way to analyze consumer behavior [7]. For example, early adopters and innovators are willing to use a novel technology product at a very early stage. Collecting responses from users and giving feedback to the new product development team is beneficial for product improvement and for attracting other users to join. 

For these reasons, usage behavior/user adoption serves as a critical factor in the adoption of IoT-based wearable fitness trackers. On the other hand, usage behavior can be considered as the ultimate measure of adoption. Venkatesh, Thong and Xu [29] indicated that the construct of usage behavior should be measured from both the aspect of variety and frequency. For technology adoption, usage behavior can be measured by four aspects [78]: (1) the amount of time spent using technology products per day, (2) the usage frequency of technology products, (3) the number of various application services used, and (4) the number of various tasks/daily activities supported technology products. In this research, usage time, usage frequency, and the number of various tasks/daily activities supported are introduced.

#### 2.3.9. Model Development

According to the literature review, the constructs and aspects of users’ adoption of IoT-based wearable fitness trackers are defined as follows: (1) perceived usability, (2) performance expectancy, (3) perceived technology utility, (4) network externality, (5) user innovativeness, (6) domain specific knowledge, (7) adoptive intention, and (8) usage behavior. These selected constructs have potentially meaningful relationships with each other and are thus regarded as major evaluation aspects. The definition of all constructs is shown in Table 1.

## 3. Materials and Methods 

**Figure 1:**
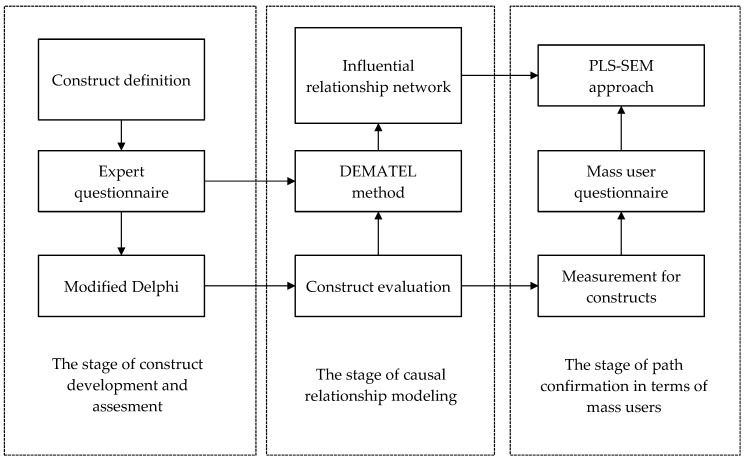
Analytical procedure of the proposed work.

The multiple criteria decision making (MCDM) method takes into consideration multiple criteria simultaneously and assists decision makers to assess the specific case and problem in terms of a small number of samples [87]. These methods have successfully been applied in various realms, including supply chain management, business, engineering, and predictions of causal relationships among criteria. In this study, the modified Delphi approach was used to evaluate the suitability of each aspect, based on experts’ consensus. Subsequently, the DEMATEL—one of the MCDM methods—was used to model a causal framework for predicting users’ adoption of IoT-based wearable fitness trackers according to the aspects derived from the literature review. To further explore the adoptive intentions and usage behaviors of mass users (general consumers) toward IoT-based wearable fitness trackers, the PLS-SEM was introduced, which is a multivariate method that integrates factor analysis and path analysis. The PLS-SEM confirms and examines the paths among variables being derived using the DEMATEL. The proposed analytical process is shown in Figure 1.

### 3.1. Data Collection

In this research, the analytical procedure consists of investigating experts’ opinions toward IoT-based wearable fitness trackers and confirmation of the opinions by mass customers. The first stage of the analytic process investigates experts’ opinion to determine the appropriateness of feasible constricts and criteria that influence technology adoption of IoT-based wearable fitness trackers. Next, selected constructs and criteria are further employed to develop a questionnaire that is sent to experts for investigation of influential relationships between variables. Based on the survey results, the DEMATEL approach was subsequently used to define the causal relationship between networks. For the first stage, 41 experts who have specific domain knowledge backgrounds in IoT-related industries, research institutions, and other fields were invited to provide opinions. These respondents were carefully selected based on their practical expertise and background. The expert survey was conducted in September 2016, via interviews with a questionnaire survey. 

To confirm the paths between the factors derived by using the DEMATEL based on experts’ viewpoints, the second stage of the research collected questionnaires based on mass users’ opinions. The questionnaire collected in the second stage encompassed items that measure the extent to which users agree with the statements related to each construct. Additionally, the items were collected according to previous studies. To effectively gather data from mass users, an online field survey was conducted in the period between 13 September and 30 October 2016. This questionnaire collected from mass users was posted on a popular website concerning the issues of IoT devices in Taiwan. To encourage participation, small monetary incentives were offered as lucky draw prizes. To reduce the possibility of someone responding to the questionnaire more than once, each respondent was required to provide basic information and an e-mail address in the survey. Later, duplicated responses, including those with similar e-mail addresses and respondent information, as well as those with incomplete responses and missing values, were deleted. Overall, 247 responses were received, and the total number of usable responses was 226. The overall response rate was 91.50%. Of the respondents, 51% were male and 49% were female. The majority of respondents (79.65%) were 20–35 years old. Additionally, 2.65% of respondents had a high school degree or less, 65.49% had an undergraduate degree, and 31.86% had a graduate degree. Furthermore, 39.82% of respondents were students, 13.27% were in manufacturing, 1.33% were in logistics, 3.10% were in finance, 17.70% were in IT, 1.77% were in healthcare, 7.08% were in the public sector or at a research institution, and 15.93% were in other occupations. The respondents reported the following usage frequencies of IoT-based wearable fitness trackers per day: less than 2 h (20.80%), 2–4 h (37.61%), 4–6 h (26.99%), and more than 7 h (14.60%). Sample demographics are shown in Appendix B.

### 3.2. Measurement Development

The questionnaire refers to previous works from which the items were adapted for this research. The items and the scales used for perceived usability and perceived technology utility were modified from Lacka and Chong [32]. The items and scales for performance expectancy of the UTAUT2 theory were adapted from Venkatesh, Thong and Xu [29]. The items and scales for network externality were adapted from [56,58]. The user innovativeness items were modified from Parasuraman [27]. The domain specific knowledge items were modified from Koo and Chung [88] as well as Han, et al. [89]. For adopting intention and usage behavior, the items belonging to adopting intention were adapted from Venkatesh, Thong and Xu [29] and Lu, et al. [90]. The items belonging to usage behavior were modified from Venkatesh, Thong and Xu [29], as well as Nikou and Bouwman [91]. Each individual was asked to indicate the extent of agreement with statements about the adoption of an IoT-based wearable fitness trackers using a five-point Likert scale on an interval level ranging from “strongly disagree” (1) to “strongly agree” (5). The main survey was conducted after determining the content validity of the questionnaire. Appendix C contains a summary of the measurement items.

### 3.3. Modified Delphi Method

Murry Jr and Hammons [92] modified the traditional Delphi technique by eliminating the first-round questionnaire that contained unstructured questions. It was simplified to replace the open-style survey. Based on this modification, the approach is commonly referred to as the modified Delphi method [93]. The modified Delphi technique is similar to the full Delphi in terms of procedure (i.e., a series of rounds with selected experts) and intent (i.e., to predict future events and to arrive at a consensus). The significant modification consists of beginning the process with a set of carefully selected items. These preselected items may be drawn from various sources including related competency profiles, synthesized reviews of the literature, and interviews with selected content experts. The primary advantages of this modification to the Delphi approach is that it typically improves the initial round response rate and provides a solid grounding in previously developed work.

### 3.4. DEMATEL Method for Establishing the Causal Relationship Framework

The DEMATEL is a kind of causal relationship method proposed by the Geneva research centre of the Battelle memorial institute [94]. The objective is to solve complex decision-making problems by means of matrix manipulation and mathematical theories. The DEMATEL method can be used to illustrate possible causal relationships belonging to specific or particular decision making problems [7,10,87]. The DEMATEL can be appropriate for analyzing the interrelations and interdependences of a limited number of samples. The detailed calculation process for the DEMATEL method, based on an earlier work by Liou and Tzeng [95], is demonstrated in Appendix A. A description of the implementation process of the DEMATEL method is briefly described as follows. First, an initial matrix based on influence relationships from one criterion to another is derived. The scale ranges from 0 to 4, indicating no influence (0), low influence (1), medium influence (2), high influence (3), and very high influence (4). Next, the initial matrix is normalized to be direct influence matrix. The third step is to attain the total influence matrix T. The final step is to calculate the sum of rows and columns. Then, an influential relationship map (IRM) is derived. The IRM model can be used to illustrate causal relationships between criteria and can offer decision makers a comprehensive structure with which to identify important criteria for determining the corresponding responses for users’ adoption of IoT-based wearable fitness trackers.

### 3.5. PLS-SEM

SEM is a salient methodology that can be leveraged to simultaneously estimate and examine a series of interrelated dependent relationships between sets of constructs. Over the past decades, the SEM has been broadly applied to various realms such as information management, market research, management science, and organization behavior. The most popular methods in SEM include two that can be used to examine path relationships: the covariance-based SEM (CB-SEM) and the variance-based PLS-SEM. Despite the differences between these two approaches, they share the same roots. For the CB-SEM, a large number of samples and an assumption of normality are required since the CB-SEM aims to minimize differences between the estimated and the sampled covariance matrices. In contrast, the PLS-SEM has more elasticity in the assumption of normality. The PLS-SEM aims to maximize the explained variance of the endogenous latent variables. To date, most of the studies in the literature suggest that the PLS-SEM can be regarded to be a replacement for CB-SEM [96,97,98]. Therefore, the PLS-SEM can be used to address complex models, explain the variance of endogenous constructs, confirm the path relationships, and develop theories in exploratory research that require a comparatively smaller sample size, non-normal distributed data, and formative measurement of latent variables [96,98]. In this research, the PLS-SEM approach is used to confirm the path relationships derived by the DEMATEL method in terms of opinions of mass users.

## 4. Results

To derive the factors influencing users’ adoption of IoT-based wearable fitness trackers, this research combines the modified Delphi, DEMATEL, and PLS-SEM methods. This section is divided into two parts: derivation of the causal model using DEMATEL and confirmation of IRM using PLS-SEM. In the following section, the industrial background and research problem are introduced. Next, the applicability of the constructs is evaluated based on the opinions provided by experts. Then, the DEMATEL method is used to derive the influence relationships between selected constructs. This is followed by two steps: use of the measurement model and structural model to validate the robustness of the model. Finally, the path relationships derived from the causal model are further confirmed using the PLS-SEM method, based on the opinions of mass users. 

### 4.1. Industrial Background and Research Gaps

In the consumer electronics market, predictions of consumer behavior are always important tasks for the marketing manager. Marketing managers must understand the reasons why consumers would like to adopt specific products. The information related to consumer behaviors is transferred to R&D departments for product development or revisions. Because of the rapid emergence and evolution of new technology, predictions of consumer behavior are especially important and difficult. IoT-based wearable fitness trackers have already influenced our daily lives. As IoT-related products emerge, considerable revenue and profits can be expected. Firms will thus launch corresponding products, services, and smart mobile appliances to attract consumers. Since there are a limited number of scholars investigating consumer behaviors related to IoT-based wearable fitness trackers, a research gap exists. Although research on technology acceptance of mobile devices is abundant, few studies have explored the adoption of IoT-based wearable fitness trackers from the viewpoint of both experts and mass users. Thus, there is a need to explore the usage behaviors and intentions of adoption toward the IoT-based wearable fitness trackers. In this research, an integrated framework is developed to analyze the adoption behavior from the perspectives of both lead users and mass customers toward the IoT-based wearable fitness trackers.

In the analyses of consumer behaviors, investigating and understanding the differences between lead users’ and mass users’ behaviors is important. According to Rogers [24], usage behaviors of lead users’ and of mass users’ are different. For novel electronic devices, such as IoT-based wearable fitness devices, electronics firms can enhance the design and marketing of these products using data from lead and mass users’ usage behavior. Therefore, collecting information on usage behavior from lead users and mass customers is essential. However, very few scholars or practitioners have attempted to do so, resulting in a research gap. To fill this gap, this research attempts to explore intention behaviors and usage behaviors of lead users and mass customers.

Moreover, most traditional approaches for exploring consumer behaviors are based on CB-SEM methods. The path relationships between constructs and variables can be derived accordingly. The exploration or confirmation of such path relationships always require sufficient samples, making such data collection time-consuming. In this work, we propose a novel approach to overcome such problems by using the DEMATEL method to derive an IRM as the path relationships and then using the PLS-SEM method to confirm the derived paths.

This research proposes a framework with which to analyze users’ adoption of IoT-based wearable fitness trackers. The empirical study is based on a smart wearable device that can be used to monitor heart rate, make transactions, and enable communications. To effectively analyze this issue, possible factors that may influence users’ adoption of smart watches were collected from the literature review. For model development, the research was implemented in Taiwan, where experts and mass users were invited to help with the investigation.

### 4.2. Suitability Evaluation of Constructs by the Modified Delphi Method

Feasible constructs were collected in the literature review. The survey was accomplished with 41 experts who have >5 years of experience in IoT-related firms and research institutions. To find suitable constructs for this research, the feasibility of constructs was evaluated with the modified Delphi method. Table 2 shows the results of this evaluation. According to the literature, agreement by experts on any particular opinion must reach a minimal consensus of 75%. The agreement rate for each construct exceeded 75%, which means that all constructs were appropriate for analyzing users’ adoption of IoT-based wearable fitness trackers.

### 4.3. Derivation of the Influential Causal Relationship by DEMATEL Method

**Figure 2:**
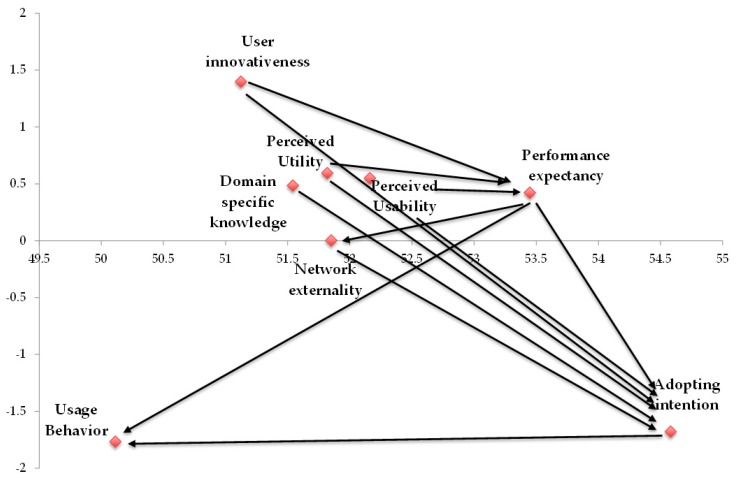
The influential causal relationship by DEMATEL method.

After the evaluation of constructs, the DEMATEL approach was used to establish an influential causal relationship. For this, four steps were followed (see Appendix A for details). First, the initial average direct-influence matrix A was built in terms of survey from 41 experts who were asked to conduct a pairwise comparison that considered the inter-influence of all constructs on each other (see Table A3 in Appendix D). To ensure the quality of the survey, the significant confidence test was utilized. The calculated value was 0.028%, which represents a significant confidence of 99.972% for the survey of experts. Additionally, Cronbach’s alpha was used to examine the survey data. The results confirmed the reliability of each construct. The reliability for all constructs ranged from 0.732 to 0.783, which met the guidance; i.e., reliability exceeded the required level of 0.70. The next step was to obtain the normalized direct-influence matrix N according to the initial matrix A using Equations (2) and (3), as shown in Table A4. Then, the total influence matrix T was obtained with Equation (4), as shown in Table A5. Based on the total influence matrix T, the prominence $r_{i}+c_{i}$ and the relation $r_{i}-c_{i}$ were obtained, which are shown in Table A6. The final step was to derive an influential causal network in terms of prominence $r_{i}+c_{i}$ and relation $r_{i}-c_{i}$ of the total influence matrix. The directions of net influence are shown in Figure 2. For any pair of constructs, only the larger influence will be demonstrated. For example, for user innovativeness (UI) and performance expectancy (PE), since the total influences from UI to PE is 3.353, while the total influences from PE to UI is 3.230, only the net influences from UI to PE are shown in Figure 2.

Concerning the “prominence $r_{i}+c_{i}$” in Table A6, adopting intention (AI) has the strongest impact on the strength of relationship ($r_{7}+c_{7}=54.577$), which indicates that adopting intention (AI) is the most important influencing construct. In contrast, usage behavior (UB) has the smallest effect ($r_{8}+c_{8}=50.111$). Regarding the “relation $r_{i}-c_{i}$”, user innovativeness (UI) has the highest degree of influence $\left(r_{2}-c_{2}=1.397\right)$. This means that user innovativeness (UI) directly influences the other constructs and is therefore the “cause”. Usage behavior (UB) has the smallest degree of influence $\left(r_{8}-c_{8}=-1.768\right)$ and was influenced the most by others and thus is the effect. The order of the other relation $r_{i}-c_{i}$ is listed as follows: the perceived technology utility (PU) $\left(r_{5}-c_{5}=0.594\right)$, perceived usability (PUS) $\left(r_{6}-c_{6}=0.549\right)$, domain specific knowledge (DK) $\left(r_{4}-c_{4}=0.484\right)$, performance expectancy (PE) $\left(r_{1}-c_{1}=0.422\right)$, network externality (NE) $\left(r_{3}-c_{3}=0.002\right)$, and adopting intention (AI) $\left(r_{7}-c_{7}=-1.679\right)$.

### 4.4. Research Hypotheses

**Figure 3:**
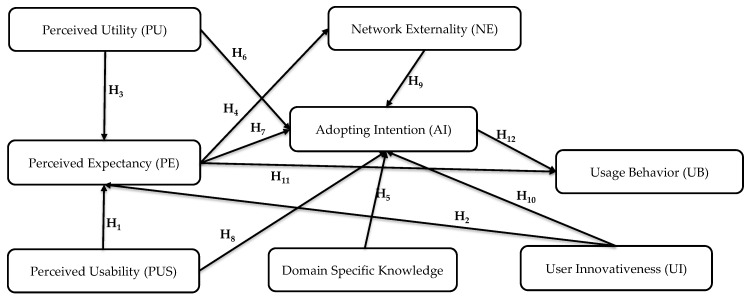
The research model. H_1_: Perceived usability has a positive effect on performance expectancy; H_2_: User innovativeness has a positive effect on performance expectancy; H_3_: Perceived utility has a positive effect on performance expectancy; H_4_: Performance expectancy has a positive effect on network externality; H_5_: Domain specific knowledge has a positive effect on adopting intention; H_6_: Perceived utility has a positive effect on adopting intention; H_7_: Performance expectancy has a positive effect on adopting intention; H_8_: Perceived usability has a positive effect on adopting intention; H_9_: Network externality has a positive effect on adopting intention; H_10_: User innovativeness has a positive effect on adopting intention; H_11_: Performance expectancy has a positive effect on usage behavior; H_12_: Adopting intention has a positive effect on usage behavior.

According to the derivation of causal relationships by the DEMATEL method, the influential paths between constructs are in Figure 3. To further confirm the path relationships, the research model based on the influential causal diagram shown in Figure 3 and the hypotheses were formed as follows: 

### 4.5. Tests of the Measurement Model.

Reliability, convergent validity, and discriminant validity of the measurement model were assessed in this study. Fornell and Larcker [99] suggested that measurement scales should be assessed using three main criteria: (1) all indicator factor loading should be significant and exceed 0.5, (2) construct reliabilities should exceed 0.8, and (3) the average variance extracted (AVE) by each construct should exceed the amount of measurement error variance (AVE > 0.5). 

Reliability analysis, comprised of Cronbach’s alpha and composite reliability (CR), was utilized to evaluate the internal consistency of the model. Nunnally [100] suggested that Cronbach’s alpha should exceed the level of 0.7. The Cronbach’s alpha of each construct obtained in this research met the guidance, ranging from 0.741 to 0.868, as shown in Table A7 in Appendix E. The CR values of all constructs were above the recommended level of 0.8, indicating adequate internal consistency. For convergent validity, all indicator loadings with reflective measures exceeded 0.5 (refer Table A7 in Appendix E). The CR values of all constructs exceeded 0.8, ranging from 0.850 to 0.919. AVE ranged from 0.59 to 0.77, which met all convergent validity conditions. Discriminant validity was evaluated based on criteria recommended by Fornell and Larcker [99]: the square root of AVE for each construct should exceed the correlation between other constructs. Table A8 shows the matrix of correlation coefficients for all constructs in this paper. Diagonal elements—the square roots of AVE from the constructs—are much larger than the correlation coefficients shared between any two constructs in the model. Additionally, in the measurement model, these constructs were necessarily different from each other. All constructs carried sufficient discriminant validity. As such, the measurement model demonstrated satisfactory reliability, convergent validity, and discriminant validity.

In addition to the measurements of the above-stated model, there was still a possibility that the whole validity of this research might be threatened by the mass user data set. Hence, Harman’s one-factor test was utilized to identify any potential common method bias [101]. The degree of harm caused by the common method bias is high if the explained variance of any single factor exceeds 50% [102]. Thus, the principle component analysis was used to detect such a bias. Based on this analysis, the largest factor explained that the 44.56% variance and accumulated explained variance was 67.75%. Thus, there was no significant common method bias in the data set. 

Furthermore, several correlation coefficients between constructs, ranging between 0.65 and 0.75 (Table A8), were rather high relative to others. This high correlation between constructs implies that multicollinearity may exist. To determine whether any multicollinearity existed, the variance inflation factors (VIF) method was leveraged. The VIF, a common measurement of multicollinearity in regression analysis, was used to indicate the degree to which one predictor variable is explained by other predictor variables. Regression analysis was employed to examine the VIF. VIF scores, ranging from 1.366 to 2.485, were less than the suggested threshold of 3.3 by Diamantopoulos and Siguaw [103]. Consequently, no significant multicollinearity existed. 

### 4.6. Tests of the Structure Model Derived by DEMATEL

**Figure 4:**
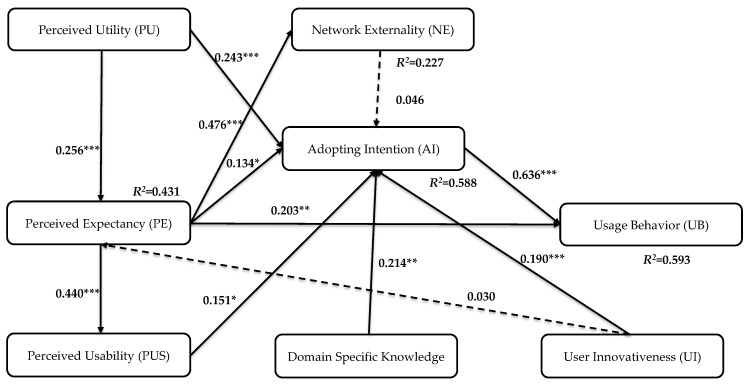
Structural model of IoT-based wearable fitness trackers adoption with path coefficients. Note: *** p<0.001, ** p<0.01, * p<0.05.

After analyzing the measurement model, the structure model derived using DEMATEL was further tested in terms of PLS-SEM analysis using the Smart PLS 2 [104]. The significance levels of the hypothesized construct relationships were estimated by applying the bootstrapping technique to 5000 bootstrap subsamples to generate t-statistics and standard errors. Figure 4 displays the path coefficients, path significances, and the variances explained by $R^{2}$ corresponding to each path. All hypotheses corresponding to the path model were supported by the analytic results, expect H_2_ and H_9_. $R^{2}$ shows that the model explains 43.05% of the variance in performance expectancy (PE), 22.68% of variance in network externality (NE), 58.85% of variance in adopting intention (AI), and 59.29% of variance in usage behavior (UB). 

This research also examined the predictive relevance by using the $q^{2}$ values [105] and measured the inner model effects between the usage behavior construct and other constructs by using the $f^{2}$ values [96]. For the $f^{2}$ values, based on the research of Cohen [106], an analytic value in the range of 0.02 to 0.15 indicates a weak effect, a value in the range of 0.15 to 0.35 indicates a moderate effect, and a value greater than 0.35 indicates a strong effect. Similar to the $f^{2}$, the $q^{2}$ was used to examine the predictive relevance. The weak, moderate, and strong degrees of predictive relevance for $q^{2}$ values were set to 0.02, 0.15, and 0.35, respectively, according to [96]. The $q^{2}$ and $f^{2}$ values associated with the path PE→UB (i.e., $f_{PE\to UB}^{2}$ and $q_{PE\to UB}^{2}$) were 0.037 and 0.069, respectively. The $q^{2}$ and $f^{2}$ values associated with the path AI→UB (i.e., $q_{AI\to UB}^{2}$ and $f_{AI\to UB}^{2}$) were 0.358 and 0.663, respectively. In addition to the confirmation of model fitness, the standardized direct, indirect, and total effects are also presented in Table 3. 

Figure 4 demonstrates the path significances. First, the PUS (β=0.440,p<0.001) and PU (β=0.256,p<0.001) had positive correlation effects with PE, whereas UI (β=0.030,p>0.05) showed a negligible direct correlation with PE. Therefore, hypotheses H_1_ and H_3_ were confirmed whereas H_2_ was not statistically significant. Second, performance expectancy (PE) (β=0.476,p<0.001) was positively correlated with network externality (NE), thereby confirming H_4_. Third, the DK (β=0.214,p<0.01), PU (β=0.243,p<0.001), PE (β=0.134,p<0.05), PUS (β=0.151,p<0.05), and UI (β=0.190,p<0.001) had direct positive correlation effects with AI, but the correlation relationship between NE and AI was not significant. Therefore, H_5_, H_6_, H_7_, H_8_, and H_10_ were statistically significant, whereas H_9_ was not. Finally, UB was directly correlated with both PE (β=0.203,p<0.01) and AI (β=0.636,p<0.001), meaning H_11_ and H_12_ were supported.

## 5. Discussion

This work developed and examined a framework regarding users’ adoption of IoT-based wearable fitness trackers. This research combines TAM-related theories and other applicable factors to evaluate why users adopt such devices. Apart from conventional methods for model establishment, this research utilized the DEMATEL method to construct an influential causal relationship framework. Then, the PLS-SEM approach was employed to confirm the path relationships. The results have important implications for practitioners, IoT application service providers, electronic firms, and researchers, who are eager to study adoption and usage of IoT-based wearable fitness trackers. In this section, the analytical results of this study are discussed from two perspectives: DEMATEL and PLS-SEM. Additionally, the combination of DEMATEL and PLS-SEM is described.

### 5.1. Predictors of Technology Adoption of IoT-Based Wearable Fitness Trackers

Influence relationships between constructs were identified by the DEMATEL method, which established the influential network relationship map. Figure 2 shows the complete influential relationships based on the opinions provided by experts. The path relationships were derived as follows. First, UI directly influences PE. Second, both PTU and PUS influence PE. Third, the AI is affected by PE, PTU, PUS, UI, DK, and NE. Finally, both PE and AI directly influence the UB. The objective of this research was to explore users’ adoption of IoT-based wearable fitness trackers. As such, the path relationships derived by the DEMATEL method (see Figure 3) were confirmed by the PLS-SEM, based on the opinions of mass users. 

PUS had a significant effect on PE for mass users, which is consistent with studies by Chuah and Rauschnabel [33]. According to these previous publications, PUS plays an important role in information systems and technology adoption. IoT technology has recently emerged and a large number of wearable fitness trackers with embedded IoT-related technologies have been launched. IoT-based wearable fitness tracker is a relatively new concept in Taiwan and people do not trust the usefulness and functionality that can be used to accomplish daily activities. Thus, with new technology embedded in wearable fitness trackers, users may expect IoT-based wearable fitness trackers to be easy to use and to serve them in a useful way. This explains the smaller effect of PU on PE relative to PUS on PE. In contrast, based on the influence relationships derived by the DEMATEL, UI influences PE-based experts’ opinions. Conversely, the PLS-SEM results revealed that there was no relationship between UI and PE. Although the studies by Hwang [107] and Leonard-Barton and Deschamps [63] showed that UI is an important determinant in new technology adoption and performance expectancy, the relationship between UI and PE was not confirmed in our research (see Figure 4). Based on Jin [108], a possible explanation is that there were significant differences in usefulness and ease of use in technology by lead users consisting of innovators and early adopters and mass users encompassing the majority of adopters and laggards. 

Second, almost all previous studies emphasized the influence of NE on PE and validated such relationships [56,58,91]. In the context of technology adoption, the install base of the product will be increased when the design of a particular product is based on several important features, including usefulness, ease of use, fashion, and utilization [109]. In our research, NE is explained by PE according to the influence relationship derived by DEMATEL and confirmed using the PLS-SEM in H_4_. These results confirm that more and more IoT-based wearable fitness trackers will become popular, which brings more value to consumers, as the number of users increase, and the network externality increases. 

Third, the relationship between PU and AI was validated in this research. This result is consistent with the findings of Lacka and Chong [32], which affirmed that users’ PU (the fit of the particular technology for achieving goals) has a direct positive effect on people’s intention to use specific social network services. Thus, this work demonstrated that if users do not perceive that a given technology generates utility, they are unlikely to use it. Similarly, two important determinants, PE and PUS, have a positive correlation with AI. This result suggests that when people perceive IoT-based wearable fitness trackers as being useful and easy to use, the degree of adopting intention will be higher. The positive relationship between PE and AI is consistent with the results of Chuah and Rauschnabel [33], which confirmed that PE has a direct positive effect on the AI of wearable technologies. Likewise, the positive relationship between PUS and AI was confirmed by Mital, Chang, Choudhary, Pani and Sun [41], which asserted that the concept of PU is a primary driver of users’ AI for cloud computing-based IoT services. The positive correlation between DK and AI was validated as being significant. Adoption of a particular technology by consumers involves various personal feelings, such as curiosity and anxiety. Awareness of related information or knowledge of a particular technology will reduce consumer anxiety. Thus, knowledge and awareness of a specific technology is a critical factor that can influence the adoption of a new technology by consumers [89]. Based on the results of PLS-SEM, UI has a significant effect on AI in the context of IoT-based wearable fitness trackers. This finding is consistent with previous TAM-based research that examined the effect of UI on intentions to use information technology [110]. Hence, consumers who have high intrinsic motivations (e.g., innovativeness) will enhance their intention to adopt IoT-based wearable fitness trackers. In addition, based on the empirical results, the NE did not have an effect (H_9_) on AI, which may be because IoT-related application services embedded in wearable fitness trackers have not yet fully been broadly generalized to our lives in Taiwan. Hence, people may not be influenced enough to adopt such devices. 

Finally, concerning the positive effects of PE and AI on UB (H_11_ and H_12_), the results imply that when people’s perception of usefulness and users’ adopting intention increase, adoption behavior will be positively influenced. The results are consistent with previous studies that showed that PE is a pivotal determinant of UB [30,111]. Similarly, the relationship between AI and UB was validated by previous studies [11,32]. Therefore, both of these two factors, which are regarded as important drivers, can effectively predict users’ adoption behavior toward IoT-based wearable fitness trackers based on the present research framework.

### 5.2. Implications for Research

This research makes three contributions. First, to the best of our knowledge, research studies of IoT-based wearable fitness trackers are scarce; thus, this study attempts to fill this research gap. Although previous studies explored adoption of smartphones and electronics devices, this paper integrated domain specific knowledge, NE, UI, PUS, and PU constructs derived from various theoretical frameworks that were adapted from the TAM-based model. This study demonstrated that the TAM and usability of technology are correlated; that is, they not only complement each other, as argued by Lacka and Chong [32], but they also correlate. Particularly, PUS is similar to the concept of ease of use in TAM. The definition of both constructs illustrates the user’s ability to use a specific technology for tackling particular activities. Likewise, the PE in the UTAUT theory refers to usefulness from the perspective of usability, as both represent the perception of whether particular activities can be accomplished by a certain technology. As such, this paper combines two models that serve as the basis for developing an analytical framework. This work leveraged the modified Delphi method to assess the feasibility of these important determinants. Then, the DEMATEL method was utilized to depict the influential causal relationship framework. Lastly, the established path relationships were examined by the PLS-SEM approach. The analytical procedure for combining DEMATEL and PLS-SEM approaches is an early attempt of analyzing usage behavior of IoT-based wearable fitness trackers. Conventional analyses for establishing the predictive model are based on a regression method or an exploratory factor analysis. Therefore, the proposed hybridization framework can be regarded as an innovative and advanced modeling technique; thus, it can be generalized to a wide range of domains for solving various research and practical issues. According to the results (see Figure 4), our model explains 58.85% of the adopting intention toward IoT-based wearable fitness trackers and 59.29% of usage behavior for IoT-based wearable fitness trackers. These findings indicate that the research model can predict users’ adopting and usage behavior toward IoT-based wearable fitness trackers and therefore is a valuable contribution to the extant body of research. 

Second, concerning the total effect on UB, the results showed that AI (0.636), PE (0.289), PU (0.229), and PUS (0.223) play key roles in influencing users’ adoption behavior toward IoT-based wearable fitness trackers. Thus, in the context of technology adoption, these determinants can be used to develop a research model. In addition, with regard to the total effect on AI, PU (0.278), DK (0.214), and PUS (0.210) had relatively strong relationships to users’ intention of adopting IoT-based wearable fitness trackers. Based on these results, these three important factors should be considered when trying to understand the intention of consumers when adopting novel technology. 

Finally, most of the previous studies on technology adoption suggested that the NE has a direct positive effect on PE. However, based on the derived theoretical framework, the path relationship was opposite; that is, PE had a positive influence on NE (H_4_). The hypothesis was validated in the present research. This finding can serve as a basis for future theoretical model development in related technology adoption research. Like other empirical studies of TAM-based analytic models, PU and PUS play an important role in shaping the AI. In addition, NE is not a significant factor in the context of IoT-based wearable fitness trackers. This is likely because IoT-based wearable fitness trackers have not penetrated the mass market. Thus, the effects of the NE are still insignificant. 

Based on results from the empirical study, this research will inspire future studies to employ similar conceptual frameworks and mixture methods to study technology adoption. Our model integrates the usability perspective, TAM model, user innovativeness, and domain specific knowledge. These constructs were demonstrated to be applicable in our model. Further, the DEMATEL is a useful modeling technique and can be utilized to model an influential framework for solving real-world problems. This paper successfully leveraged the PLS-SEM approach to further confirm the path relationship between constructs or the casual model derived by DEMATEL. In summary, the proposed model and integrative methods can be used to predict users’ adoption of IoT-based wearable fitness trackers and can be applied to a wide range of research domains of users’ adoption behaviors of novel technology.

## 6. Conclusions

This study explored usage behavior and adoption intentions of IoT-based wearable fitness trackers from two perspectives: experts (lead users) and mass customers. A conceptual framework was proposed. Factors that influenced users’ usage behavior of smart IoT-based devices were derived, and the hypothesized relationships based on the IRM derived by the DEMATEL were confirmed. In the analytic process, a conceptual framework was organized based on past works. The modified Delphi method was introduced to confirm the applicability of variables derived from the literature review results. Next, based on lead users’ perspective, the DEMATEL method was used to derive a causal relationship model depicting which path relationships can be structured. Finally, these path relationships were confirmed by using the PLS-SEM in terms of mass customers’ perspectives. In short, this research made several contributions. The following discussion summarizes the methodological and theoretical contributions.

The methodological contributions of this research are threefold. First, factors that influence users’ intentions to adopt IoT-based wearable fitness trackers were identified by using an analytic framework that can derive the dependences and influences between criteria. This work reviewed the existing literature and collected applicable aspects of PE, UI, NE, DK, PU, PUS, AI, and UB to assess the factors influencing users’ adoption behavior of smart IoT-based wearable devices. This work also adopted the modified Delphi method to confirm the suitability of collected aspects and variables in order to fit our research topic. Second, this article derived causal relationships (i.e., the IRM) by using the DEMATEL in terms of lead users’ perspectives. Compared to conventional methods used to derive path relations (e.g., the CB-SEM), the DEMATEL can be utilized to derive the IRM without large samples and statistical tests. Thus, the DEMATEL can be regarded as an alternative modeling technique to construct causal relationships. Furthermore, in order to confirm the derived IRM as the path relationships from mass customers’ perspectives, the PLS-SEM was introduced. Finally, the analytical results derived from the integrated framework consisting of the DEMATEL and the PLS-SEM method provided new insights for researchers developing future studies and guidance for practitioners and decision-makers seeking to derive strategies to enhance the adopting intentions toward IoT-based wearable fitness trackers.

This work also makes three theoretical contributions. First, research on consumer behavior related to IoT-based wearable fitness trackers is scarce. This study fills this research gap and contributes to the empirical research in this field. Second, the empirical results confirm that AI, PE, PU, and PUS play key roles in influencing consumers’ adoption behaviors toward IoT-based wearable fitness trackers. Therefore, in the context of technology adoption, these determinants should be considered when developing a research model. Lastly, by using the DEMATEL method, this research defined a new path relationship in which the PE has a direct positive correlation effect on the NE. The novel path relationship was also confirmed by using the PLS-SEM. 

Despite the valuable findings and meaningful implications provided by this study, the present research can be further improved by overcoming some critical limitations. First, the research was based on samples in Taiwan. Therefore, the generalization of the findings to other related technology adoption fields needs to be interpreted carefully. Future studies should be conducted in other countries and different districts to investigate and compare the differences with diverse antecedents to our research results. Such a comparison could be beneficial to the wearable fitness tracker industry for targeting multicultural services and global utilities. In addition, although the proposed methodologies and analytic processes were successfully validated in this study, the generalization of the methodology can be further validated using various empirical cases. Thus, future research can refer to this paper as a basis to extend and apply our proposed research methods in other research fields. For example, future research can extend our proposed method by incorporating the fuzzy theory to reduce uncertain information generation from linguistic variable transformations. Third, the current research collects data based on the opinions of both experts and mass customers, which were provided online. Such procedures cannot avoid self-selection bias. Qualitative data collected using in-depth interviews and behavior observations can be possible alternatives. Fourth, future researchers should conduct longitudinal research to examine the dynamics of users’ behaviors toward IoT-based wearable fitness trackers. Finally, some scholars (e.g., Liu and Shia [112]) questioned the rationality of the DEMATEL in over-emphasizing the influences of the indirect relationships. Liu and Shia [112] provided an external shrinkage coefficient, d, for constructing a reduced indirect relation matrix and proposed a useful validity index, Liu’s validity index, for evaluating the performance of any results derived from the DEMATEL. Future researchers should assess the generalized DEMATEL model by considering the influences of the indirect relationships. Furthermore, the influence of different stages of indirect relationships can vary. Thus, a different shrinkage factor $d^{\left(\zeta \right)}$ will be required for the indirect relationship matrix representing the influence relationships through ζ criteria (i.e., $N^{\zeta }$). As such, examining the over-emphasis of the influences of the indirect relationships in future investigators is worthwhile.

## Figures and Tables

**Table 1:** Construct definitions of users’ adoption of IoT-based wearable fitness trackers.

Constructs	Definitions
Perceived Usability	Perceived usability represents the degree to which people believe that using a technology will be free of effort [31,48]. This concept is consistent with the definition of perceived ease of use. In accordance with the study by Dwivedi, et al. [79] that indicated that perceived ease of use has a significant relationship with adoptive intentions of information technology innovation; that is, perceived usability is a dominant factor influencing adoptive intentions toward specific technologies. In this paper, perceived usability is used to explore the adoptive behaviors toward IoT-based wearable fitness trackers.
Performance Expectancy	Performance expectancy is defined as the extent to which the usage of a novel technology or product can provide benefit to consumers in performing daily activities [29]. Performance expectancy has been extensively used to analyze technology adoption. Ooi, et al. [80] stated that performance expectancy has a strong influence on the willingness of an individual to adopt a technology for improving the performance of tasks or jobs.
Perceived Utility	Perceived utility is defined as the question of whether the functionality of the system can do what is needed [31]. Perceived utility is also the primary driver that influences users to adopt an information system [81]. Therefore, such a construct can be used to assess whether a particular technology is accepted [32]. Perceived utility has been identified in a variety of technology domains such as technology acceptance [20] and technology usage [82]. This paper uses perceived utility for investigating adoptive behavior of users toward IoT-based wearable fitness trackers.
Network Externality	Network externality stands for the effect that users obtain from a product or service will contribute to more values to users with the increase of users, complementary product, or service [57]. Based on previous studies, Hsu and Lin [56] examined adoptive behavior of IoT services from the viewpoint of network externality. Li and Wang [83] investigated how network externality influenced users’ persistence in completing software systems. As previously observed, network externality is a critical factor that can be used to predict users’ adoptive behavior.
User Innovativeness	User innovativeness is the extent to which a user adopts a particular technology earlier than other people [59]. Bruner and Hensel [62] further defined user innovativeness as a risk-taking propensity exhibited by a specific group of people but not others. According to Choi and Kim [84], user innovativeness influences a wide variety of user decisions and actions and highly innovative users respond more positively to new products.
Domain Specific Knowledge	Domain specific knowledge is adapted from concept technology awareness [68]. Technology awareness stands for the users’ knowledge and understanding of a specific technology or product [69]. That is, domain specific knowledge indicates that users have sufficient knowledge and understanding to adopt a particular technology. To fit with our research, the definition of domain specific knowledge is: users that have sufficient understanding for adopting IoT-based wearable fitness trackers.
Adopting Intention	Adopting intention refers to the degree to which a person has formulated conscious plans to perform or not perform some specified future behavior(s) [76]. This concept was applied to the study of technology adoption. For example, Bruner and Hensel [85] examined cloud computing adopting intention. Obal [86] used adopting intention as a target variable to understand the possible drivers that affect this variable. Adopting intention was introduced into this research as an important factor for analyzing users’ adopting intention of IoT-based wearable fitness trackers.
Usage Behavior	Usage behavior can be considered as the ultimate measure of adoption; e.g., variety and frequency of use toward a particular technology [29]. More precisely, for technology adoption, usage behavior can be measured by four aspects [78]: (1) the amount of time spent using technology products per day, (2) the usage frequency of technology products, (3) the number of various application services used, and (4) the number of various tasks/daily activities supported by technology products. In this research, usage time, usage frequency, and the number of various tasks/daily activities supported are introduced.

**Table 2:** Results of construct evaluation using the modified Delphi method.

Title	PTU	PE	PTUS	NE	UI	DK	AI	UB
Agree	38	41	40	41	37	41	41	41
Disagree	3	0	1	0	4	0	0	0
Agree %	92.68%	100.00%	97.56%	100.00%	90.24%	100.00%	100.00%	100.00%
Disagree %	7.32%	0.00%	2.44%	0.00%	9.76%	0.00%	0.00%	0.00%

Note: PTU: perceived technology utility; PE: performance expectancy; PUTS: perceived usability; NE: network externality; UI: user innovativeness; DK: domain specific knowledge; AI: adopting intention; UB: usage behavior.

**Table 3:** The effect of constructs.

Constructs	Title	NE	PE	AI	UB
PU	Direct effects	-	0.256	0.243	-
	Indirect effects	-	-	0.034	0.229
	Total effects	-	0.256	0.278	0.229
PE	Direct effects	0.476	-	0.134	0.203
	Indirect effects	-	-	-	0.085
	Total effects	0.476	-	0.134	0.289
PUS	Direct effects	-	0.440	0.151	-
	Indirect effects	0.210	-	0.059	0.223
	Total effects	0.210	0.440	0.210	0.223
DK	Direct effects	-	-	0.214	-
	Indirect effects	-	-	-	0.121
	Total effects	-	-	0.214	0.121
UI	Direct effects	-	-	0.190	-
	Indirect effects	-	-	-	0.121
	Total effects	-	-	0.190	0.121
AI	Direct effects	-	-	-	0.636
	Indirect effects	-	-	-	-
	Total effects	-	-	-	0.636
$R^{2}$		0.227	0.431	0.588	0.593

## Appendix A. DEMATEL

The effectiveness of the DEMATEL method has been verified in a broad range of fields. It can be used to depict an influential relationship model and it also allows decision makers to understand “the important criteria/aspects” and “the extent to which one criterion influences another criterion”. The steps of the DEMATEL method are as follows.

Step 1: Establish an initial direct-influence matrix

The pair-wise comparison scale is designated with five levels, where the scores ranging from 1 to 5 stand for either no influence (0), low influence (1), medium influence (2), high influence (3), and very high influence (4). This scale is provided to experts to determine the degree of influence that relies on the interdependence between criterion i and criterion j (denoted as $a_{ij}$) for establishing a direct-influence matrix A that is an n×n matrix. To ensure the quality of the survey, a significant confidence test is introduced: $\frac{1}{n}\sum_{i=1}^{n}\sum_{j=1}^{n}\frac{\left|t_{ij}^{p}-t_{ij}^{p-1}\right|}{t_{ij}^{p}}\times 100\%$. If the derived value is less than 5%, this means that the analysis can be supported based on the significant confidence level (>95%). The significant confidence equation stands for the number of experts, $t_{ij}^{p}$ is the average influence that the i criterion has on j, and n denotes the number of criteria.

(A1) $$A=\left[\begin{array}{cccc} a_{11} & a_{12} & \cdots & a_{1n}\\ a_{21} & a_{22} & \cdots & a_{2n}\\ \vdots & \vdots & \vdots & \vdots \\ a_{n1} & a_{n2} & \cdots & a_{nn} \end{array}\right]$$

Step 2: Normalize the direct-influence matrix

The normalized direct-influence matrix can be obtained using Equations (A2).

(A2) $$z=\underset{i,j}{\min}\left\{\frac{1}{\max_{1\leq i\leq n}\sum_{j=1}^{n}a_{ij}},\frac{1}{\max_{1\leq j\leq n}\sum_{i=1}^{n}a_{ij}}\right\}$$

Step 3: Attain a total-influence matrix T

The total-influence matrix T is obtained using Equation (A3):

(A3) $$T=N+N^{2}+\cdots +N^{h}=N{\left(I-N\right)}^{-1},\;\mathrm{when}\;\underset{h\to \infty }{\lim}N^{h}={\left[0\right]}_{n\times n}$$

T is a total influence-related matrix; N is a direct influence matrix; $N={\left[x_{ij}\right]}_{n\times n}$; $\underset{h\to \infty }{\lim}\left(N^{2}+\cdots +N^{h}\right)$ stands for an indirect influence matrix; $0\leq \sum_{j=1}^{n}x_{ij}<1$ or $0\leq \sum_{i=1}^{n}x_{ij}<1$; and only one $\sum_{j=1}^{n}x_{ij}$ or $\sum_{i=1}^{n}x_{ij}$ is equal to 1 for ∀i,j. Thus, $\underset{h\to \infty }{\lim}N^{h}={\left[0\right]}_{n\times n}$. The (i,j) element $t_{ij}$ of matrix T denotes the direct and indirect influences of factor i on factor j.

Step 4: Obtain the causal relationship

The rows and columns are separately summed and denoted as $r_{i}$ and $c_{i}$ within the total-influence matrix $T=[t_{ij}]$. $r_{i}$ represents the level of direct or indirect impacts on other criteria, and $c_{i}$ represents the level to which it is affected by other criteria.

(A4) $$r={{\left[r_{i}\right]}^{\prime }}_{n\times 1}={{\left[\sum_{j=1}^{n}t_{ij}\right]}^{\prime }}_{n\times 1}={\left(r_{1},\cdots ,r_{i},\cdots ,r_{n}\right)}^{\prime }$$

(A5) $$c={\left[c_{j}\right]}_{1\times n}={\left[\sum_{i=1}^{n}t_{ij}\right]}_{1\times n}=\left(c_{1},\cdots ,c_{j},\cdots ,c_{n}\right)$$

Based on the definition, when i=j (e.g., $r_{i}+c_{i}$ represents the index that represents the strength of the influence, both dispatching and receiving), $r_{i}+c_{i}$ is the degree to which factor i plays the central role in the problem. The values of $r_{i}+c_{i}$ are placed on the x-axis and are called “prominence”. In contrast, if $r_{i}-c_{i}$ is positive, then factor i primarily influences the strength of other factors. If $r_{i}-c_{i}$ is negative, then factor i primarily receives influence from other factors. The $r_{i}+c_{i}$ values are arranged on the y-axis and are called “relation”.

## Appendix B

**Table A1:** Sample demographics.

Measurement	Item	Frequency	Percentage (%)
Gender	Male	131	57.96
	Female	95	42.04
Age	Less than 20	26	11.50
	20–35	180	79.65
	36–45	19	8.41
	More than 45	1	0.44
Education	High school or under	6	2.65
	Undergraduate	148	65.49
	Graduate	72	31.86
Occupation	Student	90	39.82
	Manufacturing	30	13.27
	Logistics	3	1.33
	Finance	7	3.10
	IT	40	17.70
	Healthcare	4	1.77
	Public sector or research institution	16	7.08
	Other	36	15.93
Frequencies using IoT-based wearable fitness trackers (per day)	Less than 2 h	47	20.80
	2–4 h	85	37.61
	4–7 h	61	26.99
	More than 7 h	33	14.60

## Appendix C

**Table A2:** Questionnaire items.

Constructs	Measurement items
Perceived usability (PTUS) (adapted from Lacka and Chong [32])	
PTUS1	The IoT-based wearable fitness trackers are easy to use for IoT services in our daily life.
PTUS2	I find it easy to get IoT-based wearable fitness trackers to do what I want them to do while accomplishing daily activities.
PTUS3	Learning to operate IoT-based wearable fitness trackers for daily activities is easy.
Performance expectancy (PE) (adapted from Venkatesh and Thong [29])	
PE1	Using IoT-based wearable fitness trackers allows me to manage daily activities in an efficient way.
PE2	Using IoT-based wearable fitness trackers makes the daily activities easier.
PE3	Using IoT-based wearable fitness trackers allow me to accomplish daily activities more quickly.
Perceived technology utility (PTU) (adapted from Lacka and Chong [32])	
PTU1	Goals of IoT-based wearable fitness trackers can be met while accomplishing daily activities.
PTU2	The features of IoT-based wearable fitness trackers enable people to effectively cope with daily activities.
PTU3	I can minimize cost with IoT-based on wearable fitness trackers while accomplishing daily activities.
Network externality (NE) (adapted from Hsu and Lin [56] and Lin and Lu [58])	
NE1	I think more and more people will adopt IoT-based wearable fitness trackers.
NE2	I think a number of relevant IoT technologies (i.e., QR code and NFC) can be used in wearable fitness trackers.
NE3	I think IoT-related devices and IoT services are very popular.
User innovativeness (UI) (adapted from Parasuraman [27])	
UI1	I learn more than others about IoT-based wearable fitness trackers.
UI2	I keep up with the latest technological developments in my area of interest.
UI3	I enjoy the challenge of figuring out how to use wearable fitness trackers for IoT application services
Domain specific knowledge (DK) (adapted from Koo and Chung [88] and Han and Wu [89])	
IK1	I agree that IoT-based wearable fitness trackers can substitute for traditional devices.
IK2	I believe that there will be more and more IoT-based service and device providers in the market.
IK3	I believe that IoT-based wearable fitness trackers are critical for our social life.
Adopting intention (AI) (adapted from Venkatesh and Thong [29] and Lu and Zhou [90])	
AI1	I intend to recommend to people that they use IoT-based wearable fitness trackers.
AI2	I have intentions of using IoT-based wearable fitness trackers in my daily life.
AI3	I am eager to use related IoT applications on my wearable fitness trackers.
Usage behavior (UB) (adapted from Venkatesh and Thong [29] and Nikou and Bouwman [91])	
UB1	I use IoT services with my wearable fitness trackers frequently.
UB2	Overall, I use IoT-based wearable fitness trackers to deal with daily activities a lot.
UB3	I spend much time using my IoT-based wearable fitness trackers.

## Appendix D. The Analytic Results of DEMATEL

**Table A3:** The direct-influence matrix A.

Aspect.	PE	UI	NE	DK	PTU	PTUS	AI	UB
Performance expectancy (PE)	0.000	2.732	2.878	2.732	2.780	3.024	3.293	2.707
User innovativeness (UI)	2.732	0.000	2.902	2.683	2.585	2.707	3.220	2.756
Network externality (NE)	2.707	2.585	0.000	2.902	2.683	2.780	3.049	2.585
Domain specific knowledge (DK)	2.683	2.805	2.951	0.000	2.683	2.707	2.976	2.561
Perceived technology utility (PU)	3.146	2.585	2.610	2.780	0.000	2.659	3.146	2.585
Perceived usability (PUS)	3.024	2.561	2.854	2.805	2.732	0.000	3.195	2.463
Adopting intention (AI)	2.805	2.585	2.659	2.634	2.805	2.732	0.000	3.585
Usage behavior (UB)	2.707	2.561	2.463	2.439	2.756	2.585	2.317	0.000

**Table A4:** The normalized direct-influence matrix N.

Aspect	PE	UI	NE	DK	PTU	PTUS	AI	UB
Performance expectancy (PE)	0.000	0.136	0.143	0.136	0.138	0.150	0.163	0.134
User innovativeness (UI)	0.136	0.000	0.144	0.133	0.128	0.134	0.160	0.137
Network externality (NE)	0.134	0.128	0.000	0.144	0.133	0.138	0.151	0.128
Domain specific knowledge (DK)	0.133	0.139	0.146	0.000	0.133	0.134	0.148	0.127
Perceived utility (PU)	0.156	0.128	0.130	0.138	0.000	0.132	0.156	0.128
Perceived usability (PUS)	0.150	0.127	0.142	0.139	0.136	0.000	0.159	0.122
Adopting intention (AI)	0.139	0.128	0.132	0.131	0.139	0.136	0.000	0.178
Usage behavior (UB)	0.134	0.127	0.122	0.121	0.137	0.128	0.115	0.000

**Table A5:** The total influence matrix T.

Aspect	PE	UI	NE	DK	PTU	PTUS	AI	UB
Performance expectancy (PE)	3.317	3.230	3.368	3.313	3.325	3.359	3.660	3.364
User innovativeness (UI)	3.353	3.033	3.288	3.231	3.237	3.266	3.569	3.285
Network externality (NE)	3.311	3.108	3.122	3.200	3.201	3.229	3.519	3.238
Domain specific knowledge (DK)	3.321	3.126	3.260	3.084	3.212	3.236	3.527	3.247
Perceived utility (PU)	3.362	3.140	3.270	3.228	3.117	3.257	3.559	3.271
Perceived usability (PUS)	3.376	3.156	3.297	3.247	3.254	3.159	3.580	3.285
Adopting intention (AI)	3.379	3.168	3.301	3.251	3.268	3.289	3.454	3.339
Usage behavior (UB)	3.095	2.904	3.019	2.974	2.995	3.011	3.261	2.912

**Table A6:** $r_{i}+c_{i}$ and $r_{i}-c_{i}$ values obtained from the total influence matrix T.

Aspect	$r_{i}$	$c_{i}$	$r_{i}+c_{i}$	$r_{i}-c_{i}$
Performance expectancy (PE)	26.936	26.514	53.449	0.422
User innovativeness (UI)	26.262	24.864	51.126	1.397
Network externality (NE)	25.926	25.925	51.851	0.002
Domain specific knowledge (DK)	26.013	25.529	51.541	0.484
Perceived utility (PU)	26.204	25.610	51.815	0.594
Perceived usability (PUS)	26.354	25.805	52.159	0.549
Adopting intention (AI)	26.449	28.128	54.577	−1.679
Usage behavior (UB)	24.171	25.940	50.111	−1.768

## Appendix E. Construct Item Statistics and the Discriminant Validity

**Table A7:** Construct item statistics.

Constructs	Items	loadings	*t*-Value	CR	AVE	Alpha
Performance expectancy	PE1	0.898	50.351	0.919	0.791	0.868
	PE2	0.885	43.573			
	PE3	0.885	46.789			
User innovativeness	UI1	0.738	15.061	0.897	0.745	0.829
	UI2	0.916	59.789			
	UI3	0.923	85.175			
Network externality	NE1	0.819	32.310	0.889	0.727	0.813
	NE2	0.891	42.389			
	NE3	0.846	24.091			
Domain specific knowledge	DK1	0.801	22.422	0.850	0.655	0.741
	DK2	0.820	32.348			
	DK3	0.806	25.495			
Perceived utility	PU1	0.826	39.128	0.888	0.726	0.811
	PU2	0.859	37.660			
	PU3	0.870	45.090			
Perceived usability	PUS1	0.876	52.665	0.907	0.764	0.846
	PUS2	0.867	39.758			
	PUS3	0.880	44.952			
Adopting intention	AI1	0.853	33.501	0.903	0.756	0.840
	AI2	0.886	46.537			
	AI3	0.871	35.921			
Usage behavior	UB1	0.888	47.945	0.897	0.745	0.828
	UB2	0.890	48.590			
	UB3	0.809	26.488			

**Table A8:** The discriminant validity of this research.

Constructs	AI	IK	NE	PE	PTU	PTUS	UA	UI
Adopting intention (AI)	0.870							
Domain specific knowledge (DK)	0.652	0.809						
Network externality (NE)	0.494	0.621	0.853					
Performance expectancy (PE)	0.571	0.579	0.476	0.889				
Perceived utility (PU)	0.655	0.639	0.442	0.567	0.852			
Perceived usability (PUS)	0.633	0.628	0.497	0.626	0.677	0.874		
Usage behavior (UB)	0.752	0.561	0.429	0.566	0.674	0.625	0.863	
User innovativeness (UI)	0.497	0.426	0.360	0.308	0.400	0.398	0.457	0.863
